# The effect of breathing exercises and mindset with or without cold exposure on mental and physical health in persons with a spinal cord injury—Results of a randomized controlled trial

**DOI:** 10.1177/02692155261428705

**Published:** 2026-03-11

**Authors:** Sonja de Groot, Frank WL Ettema, Max van der Bijll, Thomas WJ Janssen, Wendy J Achterberg-Warmer, WHM study group, Sven P Hoekstra

**Affiliations:** 1 Center of Excellence for Rehabilitation Medicine, 1190 University Medical Center Utrecht Brain Center, University Medical Center Utrecht and De Hoogstraat Rehabilitation, Utrecht, the Netherlands; 2 Department of Human Movement Sciences, Faculty of Behavioural and Movement Sciences, Vrije Universiteit Amsterdam, Amsterdam Movement Sciences, The Netherlands; 3 Amsterdam Rehabilitation Research Center Reade, Amsterdam, The Netherlands; 4 84706 Reade Center for Rehabilitation & Rheumatology, Amsterdam, The Netherlands; 5 Department of Exercise and Sport Science, 7076 St Mary’s University, San Antonio, TX, USA; 6 School of Sport, Exercise and Health Sciences, Loughborough University, Loughborough, UK

**Keywords:** Breathing Exercises* / methods, Cold Temperature*, Health Status, Mental Health, Spinal Cord Injuries* / complications, Spinal Cord Injuries* / rehabilitation

## Abstract

**Objective:**

To examine the effects of the Wim Hof Method (WHM), with cold exposure (WHM-C) and without (WHM-NC), on mental and physical health in individuals with chronic spinal cord injury (SCI).

**Design:**

Randomized-controlled trial.

**Setting:**

Rehabilitation center.

**Participants:**

Sixty-two adults with chronic SCI (37% tetraplegia, 41% motor complete) were randomized into three groups: WHM-C, WHM-NC, and usual care (UC).

**Intervention:**

The intervention groups participated in a 7-week program involving weekly supervised sessions and daily home practice. WHM-NC included breathing exercises and mindset training, while WHM-C also incorporated cold exposure. UC participants received no intervention.

**Main measures:**

The primary outcome was the Mental Health Inventory (MHI-5) score. Secondary outcomes included inflammatory and metabolic markers, pulmonary function, body composition, sleep quality, spasticity, chronic pain, and psychological stress.

**Results:**

WHM-NC had eight dropouts, mostly due to motivation or unrelated medical issues, while WHM-C and UC had one dropout each. No significant differences in MHI-5 scores were observed between groups. However, WHM-C participants reported reduced pain interference in daily activities compared to UC (p=0.027). WHM-C also showed improvements in inspiratory parameters FIV1 (+27%, p=0.02) and PIF (+23%, p=0.02) and a significant reduction in fasting glucose concentration (-6%, p=0.006) compared to UC.

**Conclusions:**

While 44% of WHM-NC dropped out, WHM-C appears to be a feasible, effective intervention for improving aspects of physical health and pain perception in a broad group of individuals with chronic SCI, although no significant mental health benefits were observed.

## Introduction

A spinal cord injury can lead to a variety of secondary complications, such as respiratory problems due to decreased respiratory function, spasticity, chronic pain,^1^ chronic low-grade inflammation,^2^ and unfavorable body composition and lipid profile.^3^ Besides physical complications, persons with spinal cord injury often experience psychological difficulties, such as a high perceived stress, which is directly related to depression, anxiety, and reduced life satisfaction.^4^ These complications are often persistent and multifactorial, requiring interventions that address both physiological and psychological domains simultaneously.

The Wim Hof Method may have a positive effect on these physical and psychological secondary complications and offers a unique integrative approach that is both accessible and low-cost, making it particularly suitable for individuals with chronic spinal cord injury who may face barriers to conventional therapies. The Wim Hof Method is based on three elements: 1) deep breathing exercises; 2) mindset, that is, commitment to maintaining focus on the task without distraction; and 3) gradual cold exposure (e.g., cold showers or cold-water immersion).^5^ The development of the Wim Hof Method and its content is described in detail in a systematic review.^6^

The effects of the Wim Hof Method breathing and mindset exercises were previously investigated in persons with spinal cord injury,^7^ showing that this intervention is feasible and can lead to improvements in respiratory function and sleep outcomes. These results are supported by studies investigating the effect of the Wim Hof Method in able-bodied and other patient groups, suggesting that the intervention may reduce inflammation in healthy adults and those with axial spondyloarthritis.^6^ Additional benefits have been reported on psychological response such as perceived stress, well-being, and depression.^6,8^ However, not all studies have found positive effects; for example, one study showed that daily practice of the Wim Hof Method did not improve cardiovascular or psychological parameters.^9^

Existing studies on the Wim Hof Method have limitations, including lack of randomization,^7,8^ short intervention durations,^8,9^ and not including cold exposure.^7^ Cold exposure and breathing exercises may offer independent benefits—cold exposure improves insulin sensitivity,^10^ lipid profiles,^11^ mood and depression,^12^ while breathing exercises reduce stress and anxiety^13^—yet direct comparisons between interventions remain lacking. To address these gaps, a randomized controlled trial with a larger sample size comparing the Wim Hof Method with and without cold exposure in persons with spinal cord injury is needed to obtain a more robust evidence base for the clinical application of this intervention.

The present study, therefore, investigated the effects of a 7-week Wim Hof Method intervention, with and without cold exposure, on both mental and physical health outcomes in persons with chronic spinal cord injury through a three-arm randomized controlled trial design. The primary outcome was mental health, while secondary outcomes included inflammatory and metabolic markers, pulmonary function, body composition, sleep quality, spasticity, chronic pain, and psychological stress. It was hypothesized that the Wim Hof Method with cold exposure would lead to greater improvements in mental health and secondary outcomes compared to the method without cold exposure, and that both interventions would outperform usual care.

## Methods

The study protocol was previously described in a dedicated protocol paper^14^ and registered at ClinicalTrials.gov (Trial registration number: NCT05704322). We adhered to the CONsolidated Standards Of Reporting Trials (CONSORT) 2025 checklist for reporting randomized trials.^15^

Ethical approval was obtained from the Medical Ethics Committee of the Máxima Medical Centre (Veldhoven, the Netherlands; identifier: w22.069), in accordance with the Helsinki Declaration revised in 1983, and informed consent was signed by all participants prior to any study procedures.

### Participants

Participants were actively recruited during outpatient rehabilitation visits at Reade Center for Rehabilitation & Rheumatology (Amsterdam, the Netherlands), and passively recruited via the Dutch spinal cord injury patient organization and other rehabilitation centers with a spinal cord injury unit. Screening and enrollment were conducted by rehabilitation professional.

The participants’ inclusion criteria were as follows: having a chronic spinal cord injury (time since injury ≥ 1 year), regardless of level or completeness—assessed using the ASIA scale—and being aged between 18 and 75 years.

Exclusion criteria were: a history of severe autonomic dysreflexia, insufficient mastery of the Dutch language, severe cognitive or communicative disorders, cardiac arrhythmias or disease, progressive disease, being or becoming pregnant during the study period, severe psychiatric illness or disorders, severe pulmonary disease, recent experience with (parts of) the Wim Hof Method that may have resulted in improvements in outcome measures, being involved in another intervention which may have an effect on the outcome measures of the present study, and contraindication identified by a physician to participate in the intervention based on a medical screening.

### Study Design

A three-arm, parallel-group, superiority randomized controlled trial was conducted to compare two intervention groups (Wim Hof Method with and without cold exposure) and a usual care group. Following pretreatment assessments, participants in the Wim Hof Method with and without cold exposure groups engaged in a 7-week intervention, while those allocated to usual care were asked to continue their daily life as usual. Posttreatment assessments were made 3–10 days following the final Wim Hof Method group session. Participants continued with the home-based sessions of the intervention until the posttreatment assessment.

### Randomization, Treatment Allocation, and Blinding

After enrollment, participants were randomized in a 1:1:1 allocation ratio to one of three study groups. Randomization was stratified by level of injury (tetraplegia/paraplegia) using a web-based program (studyrandomizer.com) and a random permuted block algorithm with variable (6–12) block sizes. The randomization sequence was generated and managed by the Principal Investigator (SdeG), who was not involved in participant enrollment or intervention delivery.

Blinding of participants to their group allocation was difficult to accomplish due to the obvious differences between conditions. However, to minimize expectancy effects and motivational bias, no explicit mention of expected intervention benefits was communicated by the investigators. To enhance engagement, participants allocated to usual care were offered the Wim Hof Method intervention after the posttreatment assessments. Pre- and posttreatment assessments were performed by independent assessors. Although the study design aimed to ensure blinded assessment, blinding could not be fully maintained during data collection, as participants occasionally and inadvertently revealed their group allocation.

### Intervention Groups

The Wim Hof Method is structured around three core components: breathing exercises, mindset, and cold exposure. Participants in the Wim Hof Method without cold exposure engaged in breathing exercises and mindset only, while those in the Wim Hof Method with cold exposure group additionally took cold showers. Detailed instructions were provided at the start of the intervention. Both intervention groups participated in weekly group sessions led by a certified Wim Hof Method instructor and were instructed to perform one daily Wim Hof Method session at home. To facilitate communication and support, participants were in a WhatsApp group with the therapists/instructors, allowing them to ask questions about their home sessions.

#### Breathing Exercises

The Wim Hof Method breathing protocol consisted of two exercises. First, participants performed deep inhalations through the nose or mouth, followed by relaxed exhalations through the mouth, for an average of 30 breaths. After the final exhalation, the participant exhaled deeply and held their breath in an unforced manner until they felt a stimulus to inhale (“retention phase”). For safety reasons, breath retention was limited to a maximum of 3.5 min. This was followed by a deep inhalation breath, held for 15 s, after which a new cycle began. Participants followed the breathing instructions—that is, when to inhale and exhale—provided by the voice guidance in the Wim Hof Method app. During the first 3 weeks, participants completed 3 cycles of 30 breaths per session, and 4 cycles of 40 breaths thereafter.

#### Mindset

The mindset was trained during the breathing exercises in both intervention groups, and in the cold exposure group, it was also trained during the cold showers, that is, to focus on their breathing rather than on the cold. There was no additional time dedicated specifically to mindset training. Commitment to focusing on the task without being distracted was encouraged during the group sessions and required during the home-based sessions. Key psychological components of the Wim Hof Method, that is, willpower, self-control, and commitment, were emphasized as essential to the practice.

#### Gradual Cold Exposure

Throughout the intervention, the participants of the Wim Hof Method with cold exposure underwent progressive cold exposure. During the first 14 days, cold exposure—specifically adapted for our group with SCI—consisted of washing nonparalyzed body parts with cold cloths. Once tolerated, the paralyzed body parts were washed similarly. After this initial phase, the regular progressive cold-exposure component of the Wim Hof Method was introduced. Participants began taking cold showers starting at 30 s, increased by 10 s each day until reaching a maximum of 2.5 min. Participants were asked to measure the temperature of the water with a thermometer (Digital thermometer, Mini LCD - TH011) they received from the researchers. The first cold shower was taken under supervision of a health care professional at the rehabilitation center. After the final group session, participants in the Wim Hof Method with cold exposure group were offered the opportunity to take an ice bath for a maximum of 2 min.

### Usual Care Group

Participants in the usual care group participated only in the pre- and posttreatment assessments and were requested to continue their daily life as usual during the 7 weeks in between.

### Outcome Measures

The following questionnaires and tests were administered pre- and post-treatment (after 7 weeks, i.e., immediately following the intervention period for the intervention groups).

#### Primary Outcome

Mental health was assessed through the Mental Health Index-5, a subscale of the 36-Item Short Form Health Survey (SF-36).^16^ The Mental Health Index-5 specifically assesses five aspects of mental health: nervousness, sadness, peacefulness, mood, and happiness. A sum score was calculated ranging from 0 (lowest mental health) to 100 (highest mental health). A cutoff point of ≤72 refers to mental health problems, and a cutoff point of ≤60 refers to severe mental health problems.

#### Psychological Outcomes

Health-related quality of life was assessed with the Dutch translation of the mental and physical component of the SF-36.^17^ The SF-36 comprises 36 items and evaluates eight domains: physical functioning, role limitations due to physical health, bodily pain, general health perceptions, vitality, social functioning, role limitations due to emotional problems, and general mental health. Each domain score, including the Mental Health Index-5, ranges from 0 to 100, with higher scores indicating better health-related quality of life.

Mindful attention awareness was measured with the 16-item Mindful Attention Awareness Scale.^18^ The average score is low (0–3.58), meaning a low self-reflection, behavior, and/or environmental awareness; moderate (3.58–4.22), meaning varying degrees of awareness; or high (above 4.22), meaning a lot of awareness.

Experienced hyperventilation was assessed with the 16-item Nijmegen Hyperventilation Syndrome Questionnaire.^19^ The Nijmegen Hyperventilation Syndrome questionnaire primarily focuses on the subjective and psychological aspects of breathing and how it reacts to stress. A total score exceeding 23 indicates notable hyperventilation symptoms.

The 9-item Pittsburgh Sleep Quality Index^20^ was used to assess sleep patterns and perceived sleep quality over the preceding month. The Pittsburgh Sleep Quality Index consist of the component scores subjective sleep quality, sleep latency, sleep duration, habitual sleep efficiency, sleep disturbances, use of sleep medication, and daytime dysfunction. The overall Pittsburgh Sleep Quality Index score is derived from the sum of these seven component scores, with lower total scores indicating better sleep quality.

Chronic pain was measured with the International Spinal Cord Injury Pain Basic Data Set,^21^ including items related to pain intensity, interference with daily activities, mood, and sleep during the last week.

The degree of spasticity of the legs at the test day and the degree of discomfort due to this spasticity were scored on a 0–10 scale. The hindrance participants perceive due to spasticity was assessed for different aspects of daily living activities: sleeping, making transfers, washing and clothing, wheelchair maneuvering and propulsion, and “other activities.”^22^ An overall spasticity hindrance score was calculated by summing the individual scores, yielding a total score ranging from 0 (no hindrance) to 10 (significant hindrance across all activities of daily living).

#### Physical Health Outcomes

Pulmonary function was assessed according to a standardized protocol^23^ with the EasyOne Air (NDD Medical Technologies, Andover, MA, USA). The following parameters were measured: forced vital capacity, forced expiratory volume in 1 s, forced inspiratory volume in 1 s, peak expiratory flow and peak inspiratory flow. Participants had to breathe through a mouthpiece while wearing a nose clip. Each measurement was performed until 3 reproducible measurements within at least ± 5% were registered. The highest measured value of each parameter was used for further analysis.

Blood pressure was assessed in triplicate through an automated arterial pressure monitor (Omron M6 Comfort (HEM-7360-E), Omron Healthcare, Kyoto, Japan).

Body mass was measured to the nearest 0.1 kg on a wheelchair weighing scale (SECA Benelux, Medical Measuring Systems and Scales, Naarden, the Netherlands). Mass from the wheelchair was deducted from the total measured mass (wheelchair + user) to determine the body mass of the individual.

Body composition was assessed in a supine position by bioimpedance analysis (Bodystat 1500MDD, Bodystat Inc, Douglas, United Kingdom). Two electrodes were attached to the right foot, at the end of the second metatarsal, and between the medial and lateral malleoli of the ankle. Two other electrodes were attached to the right hand, at the distal end of the third metacarpal and between the styloid processes of the radius and ulna. The participants were asked not to participate in heavy exercise and to empty the bladder before the measurement. Furthermore, the participant was asked to lie down quietly for a few minutes before the measurement started so that fluid could evenly be distributed over the body. Outcome measures of BIA are fat mass (kg) and lean body mass (kg).

Following an overnight fast and 15 min of seated rest, a blood sample was collected from an antecubital vein. The circulating concentration of C-reactive protein, glucose, triglycerides, total cholesterol, and low- and high-density lipoprotein-cholesterol was determined immediately following blood draw by a specialized laboratory facility at the Amsterdam University Medical Center. At the same laboratory facility, plasma was stored at −80 °C for batch analysis of interleukin-6 concentration via enzyme-linked immunosorbent assays.

#### Adherence and Experiences

All adverse events and serious adverse events were monitored throughout the study period. Participants were asked to complete a diary after each Wim Hof Method session between the first and last group session, noting the number of breaths and cycles per session, their subjective experience, and any cold exposure, including duration and water temperature, and any unexpected symptoms or complications, if applicable. These diaries were reviewed by the study team during each weekly group session and used to assess adherence to the intervention. Finally, an in-person exit interview was conducted to gather qualitative data on participants’ experiences with the intervention.

### Sample Size

The sample size calculation of this study was performed a priori on the main outcome measure Mental Health Inventory-5.^17^ Based on minimum clinically important difference of the Mental Health Index-5,^24^ Mental Health Index-5 data of the Wim Hof Method pilot study,^7^ and a 10% drop-out rate, resulted in a required sample size of 20 participants per group.^14^ For a detailed description of the sample size calculation, we refer to our study protocol paper.^14^

### Statistical Analyses

The 25^th^ version of SPSS was used for all statistical analyses. Potential differences in baseline characteristics among the three groups were assessed using a chi-square test and one-way analysis of variance. To examine whether participants who dropped out of the study differed significantly in personal and lesion characteristics from those who completed the study, independent *t*-tests, and chi-square tests were used.

To evaluate intervention effects, a linear regression analysis was performed with the difference in change of the outcome measures between pre- and posttreatment assessments as the dependent variable. Group allocation was included as independent variable, using two dummy variables: one with usual care as the reference (i.e., usual care vs. Wim Hof Method with cold exposure, and usual care vs. Wim Hof Method without cold exposure) and another, in a second regression analysis, with Wim Hof Method without cold exposure as the reference (i.e., Wim Hof method without cold exposure vs. usual care, and Wim Hof method without cold exposure vs. Wim Hof method with cold exposure). Participants who dropped out during the study were included in the study based on the intention-to-treat principle. Since low values of C-reactive protein and interleukin-6 were reported as <0.3 or <0.6 (C-reactive protein) and <1.50 (interleukin-6), and entered into the database as 0.29, 0.59, and 1.49, respectively, and given the presence of some outliers (> mean ± 3 × standard deviation), sensitivity analyses were conducted with these data points excluded.

Adherence was defined as the number (and percentage) of prescribed sessions completed between the first and last group session. A frequency table was created to summarize the participants’ reported experiences.

## Results

**Figure 1.**
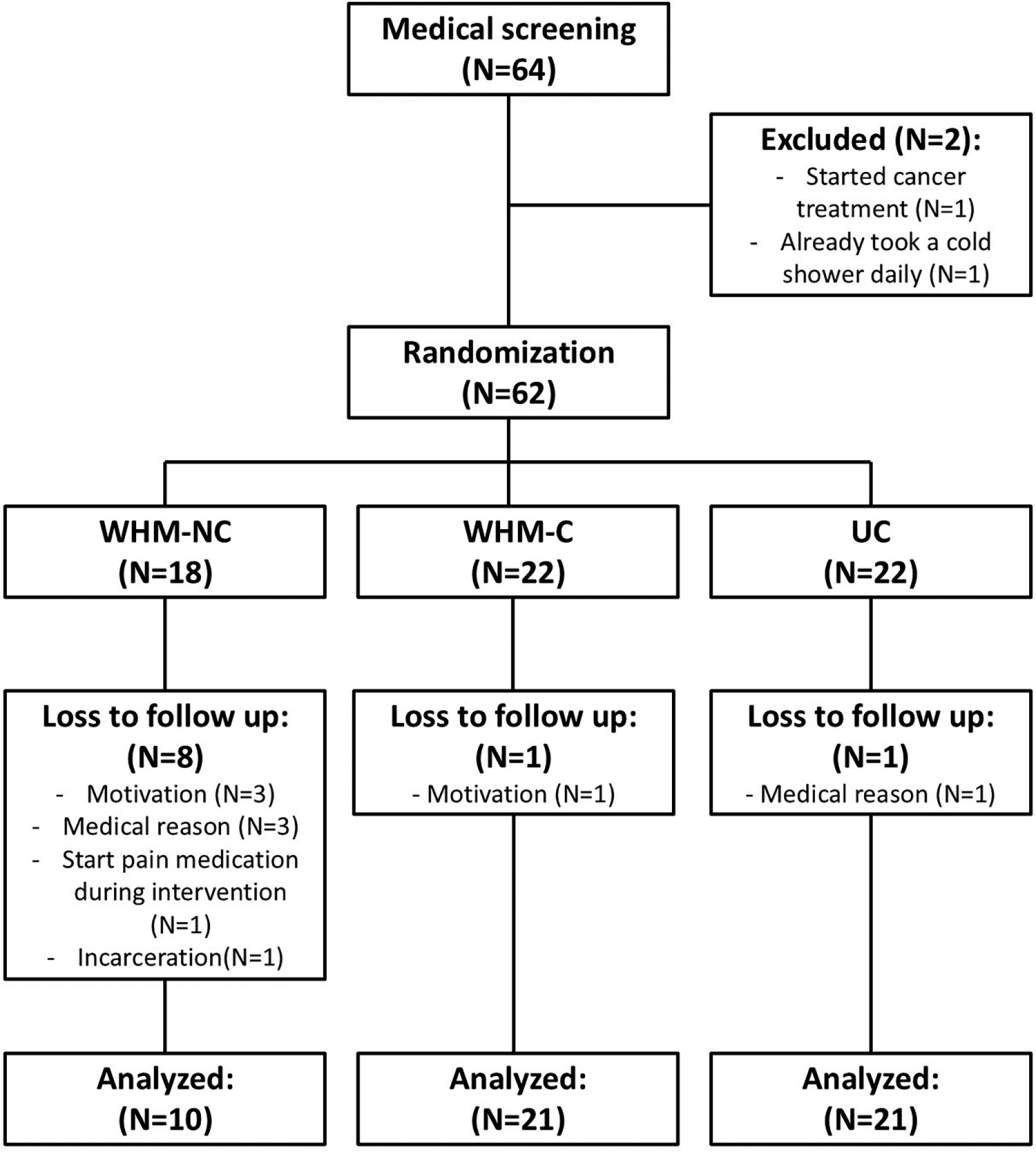
Flowchart of participants through the trial.

Participant enrolment commenced March 2023, and the last participant completed the posttreatment assessment in December 2024. The flowchart in Figure 1 outlines participant numbers from initial screening to final data analysis and describes the reasons for dropout. The medical reasons were unrelated to the intervention. The participants who dropped out of the study did not show significantly different personal or lesion characteristics compared to the total group, nor did the Wim Hof Method without cold exposure participants who did and did not drop out of the study differ significantly (Table 1).

**Table 1.** Personal and lesion characteristics at baseline.

	Total group	Usual Care	WHM-No Cold	WHM-Cold	*p*-value	Drop-outs
N	52	21	10	21		10
Male sex, *N* (%)	30 (59%)	11 (52%)	7 (70%)	13 (62%)	0.622^a^	8 (80%)
Age (years)	55.6 ± 10.4	55.9 ± 10.7	52.2 ± 6.1	57.0 ± 11.5	0.475^b^	53.7 ± 15.2
Lesion level						
Tetraplegia, *N* (%)	19 (37%)	9 (43%)	3 (30%)	7 (33%)	0.727^a^	5 (50%)
Paraplegia, *N* (%)	32 (63%)	12 (57%)	7 (70%)	14 (67%)		5 (50%)
AIS						
A, *N* (%)	13 (25%)	3 (14%)	3 (30%)	8 (38%)	0.372^a^	5 (50%)
B, *N* (%)	8 (16%)	4 (19%)	1 (10%)	3 (14%)		0 (0%)
C, *N* (%)	5 (10%)	3 (14%)	2 (20%)	0 (0%)		1 (10%)
D, *N* (%)	25 (49%)	11 (53%)	4 (40%)	10 (48%)		4 (40%)
Time since injury (years)	17.3 ± 14.9	14.9 ± 15.5	20.1 ± 12.2	19.0 ± 15.6	0.569^b^	15.6 ± 14.6
Cause of injury						
Nontraumatic, *N* (%)	24 (47%)	11 (52%)	5 (50%)	9 (43%)	0.819^a^	3 (30%)
Traumatic, *N* (%)	27 (53%)	10 (48%)	5 (50%)	12 (57%)		7 (70%)

WHM: Wim Hof Method; *N*: number of participants; AIS grades A through D describe the degree of motor and sensory function preserved below the level of injury, with A indicating the most severe impairment and D the least severe.

a Chi-square test.

b One-way ANOVA.

Three participants in the Wim Hof Method with cold exposure group partially discontinued the intervention due to adverse experiences, but completed posttreatment assessments. One participant stopped the breathing exercises after 2 weeks—due to painful tingling sensation in his legs—but continued with cold exposure; another discontinued the home exercises after 5 weeks due to pain and sleep disturbances; and a third participant withdrew halfway through the intervention due to pain that was already present prior to the intervention.

The groups that participated in both the pre- and posttreatment assessments did not differ in personal and lesion characteristics (Table 1). Since the two Wim Hof Method intervention groups did not show significant differences in their changes over time, Tables 2 and 3 present only the linear regression results using the usual care group as the reference. The reported average water temperature of the cold showers at home was 14.6 °C ± 3.6 °C.

**Table 2.** Results of the regression analyses for the psychological outcomes.

	Absolute values							Outcome regression analysis of difference		
	Usual Care *N* = 21		WHM-No Cold *N* = 10		WHM-Cold *N* = 21			UC	UC vs. WHM-No Cold	UC vs. WHM-Cold
	Pre	Post	Pre	Post	Pre	Post		Constant	β (SE)	β (SE)
*SF-36 subscales*										
MHI-5	76.2 (13.8)	76.0 (15.0)	71.6 (13.4)	74.8 (10.3)	75.1 (12.3)	72.4 (14.8)		−.190 (2.357)	3.390 (4.150)	−2.476 (3.333)
Physical functioning	36.9 (25.5)	40.2 (28.9)	32.0 (28.1)	36.0 (32.6)	37.1 (28.5)	39.3 (32.0)		3.333 (4.175)	.667 (7.351)	−1.190 (5.904)
Role limitations physical health	39.3 (38.4)	40.5 (42.9)	42.5 (44.2)	47.5 (43.2)	50.0 (41.1)	60.7 (40.0)		1.190 (9.011)	3.810 (15.87)	9.524 (12.74)
Bodily pain	57.2 (24.5)	60.2 (21.6)	42.4 (25.1)	49.7 (22.0)	63.4 (24.3)	70.0 (28.2)		3.048 (3.879)	4.252 (6.829)	3.524 (5.485)
General health perceptions	51.9 (19.7)	56.2 (20.4)	44.0 (17.3)	44.0 (17.8)	52.6 (16.0)	58.6 (20.3)		4.286 (2.342)	−4.286 (4.124)	1.667 (3.312)
Vitality	60.0 (17.8)	56.4 (14.2)	54.0 (9.7)	57.0 (13.0)	59.3 (17.6)	62.9 (14.9)		−3.571 (2.986)	6.571 (5.257)	7.143 (4.223)
Social functioning	69.2 (22.2)	65.7 (21.6)	60.3 (24.9)	67.7 (22.3)	72.9 (19.5)	75.8 (22.4)		−3.571 (4.162)	10.971 (7.328)	6.524 (5.886)
Role limitations emotional problems	77.8 (37.0)	74.1 (32.7)	80.0 (42.2)	83.4 (32.4)	74.6 (37.9)	82.5 (37.5)		6.333 (6.718)	−2.933 (11.83)	1.571 (9.501)
Health change	63.1 (25.8)	65.5 (24.3)	40.0 (17.5)	50.0 (20.4)	56.0 (26.1)	59.5 (29.0)		2.381 (4.596)	7.619 (8.093)	1.190 (6.500)
Mindful (MAAS)	4.2 (0.5)	4.4 (0.7)	4.1 (0.6)	4.2 (0.6)	4.1 (1.0)	4.1 (0.9)		.190 (.113)	−.090 (.199)	−.143 (.160)
Hyperventilation (NHSQ)	17.0 (7.9)	16.7 (7.0)	17.4 (9.0)	16.8 (8.9)	16.9 (13.1)	18.1 (13.6)		−.286 (.976)	−.314 (1.719)	1.571 (1.381)
PSQI	6.6 (2.6)	6.8 (3.1)	7.6 (2.9)	7.30 (3.2)	7.5 (3.2)	7.1 (3.9)		.190 (.479)	−.490 (.844)	−.571 (.678)
*Chronic pain*										
Intensity	6.4 (1.9)	5.8 (1.7)	6.5 (2.2)	6.2 (2.0)	5.6 (2.0)	5.2 (1.9)		−.571 (.299)	.271 (.463)	.238 (.478)
Interference ADL	3.5 (2.5)	4.0 (2.7)	6.1 (3.1)	5.7 (3.1)	3.6 (3.0)	2.6 (2.9)		.571 (.477)	−.971 (.841)	**−1.524** (.675)*
Interference mood	3.2 (2.2)	3.1 (2.4)	4.7 (1.8)	5.3 (1.5)	3.1 (2.9)	2.3 (2.8)		−.095 (.438)	.695 (.771)	−.714 (.619)
Interference sleep	5.4 (3.1)	5.4 (3.0)	6.4 (3.1)	6.9 (3.0)	4.7 (3.8)	4.1 (3.4)		−.00001 (.473)	.500 (.832)	−.524 (.668)
*Spasticity*										
Degree	3.2 (3.3)	2.5 (2.8)	3.7 (3.3)	3.2 (3.3)	2.7 (3.0)	1.9 (2.6)		−.667 (.346)	.167 (.609)	−.095 (.489)
Degree of Discomfort	2.9 (3.0)	2.4 (2.9)	3.3 (2.8)	2.8 (2.6)	1.6 (2.7)	1.6 (2.5)		−.429 (.410)	−.071 (.721)	.381 (.579)
Hindrance spasticity	2.5 (2.9)	2.0 (2.8)	2.5 (2.4)	2.0 (2.9)	1.2 (1.9)	1.3 (1.9)		−.476 (.260)	−.024 (.458)	.571 (.368)

WHM: Wim Hof Method; UC: usual care; *N*: number of participants; β: beta coefficient; SE: standard error; Pre: pretreatment assessment; Post: posttreatment assessment; MHI-5: Mental Health Index-5; SF-36: 36-Item Short Form Health Survey; MAAS: Mindful Attention Awareness Scale; NHSQ: Nijmegen Hyperventilation Syndrome Questionnaire; PSQI: Pittsburgh Sleep Quality Index; ADL: activities of daily living.

* The values in bold and marked with asterisks indicate the significantly different changes over time between the two groups.

**Table 3.** Results of the regression analyses for the physical health outcomes.

	Absolute values							Outcome regression analysis on difference pre-post		
	Usual Care (*N* = 21)		WHM-No Cold (*N* = 10)		WHM-Cold (*N* = 20)			UC	UC vs. WHM-No Cold	UC vs. WHM-Cold
	Pre	Post	Pre	Post	Pre	Post		Constant	β (SE)	β (SE)
*Respiratory function*										
FVC (L)	3.65 (0.89)	3.76 (1.00)	4.05 (0.67)	4.04 (0.66)	3.62 (1.34)	3.72 (1.36)		.109 (0.057)	−.116 (.101)	−.004 (0.081)
FEV-1 (L)	2.83 (0.81)	2.89 (0.84)	3.12 (0.67)	3.18 (0.57)	2.81 (0.92)	2.89 (0.96)		0.052 (0.050)	0.014 (0.087)	0.023 (0.070)
FIV-1 (L)	2.65 (1.27)	2.50 (1.51)	2.84 (1.11)	3.01 (0.69)	2.34 (1.58)	2.98 (1.37)		−.154 (.234)	.329 (.412)	**.792** (.331)*
PEF (L/sec)	6.84 (2.59)	7.19 (2.71)	7.71 (1.50)	8.37 (1.60)	7.20 (2.56)	7.46 (2.25)		.346 (.194)	.320 (.341)	−.083 (.274)
PIF (L/sec)	4.49 (2.31)	4.35 (2.36)	4.88 (2.21)	5.55 (1.82)	3.79 (1.89)	4.68 (1.43)		−.135 (.312)	.801 (.549)	**1.023** (.441)*
*Blood pressure*										
Systolic BP (mm Hg)	121 (22)	125 (21)	125 (26)	122 (25)	127 (22)	125 (21)		3.810 (2.931)	−7.510 (5.160)	−5.524 (4.145)
Diastolic BP (mm Hg)	77 (15)	79 (12)	76 (13)	75 (14)	78 (11)	77 (11)		1.905 (1.704)	−2.805 (3.001)	−2.762 (2.410)
*Body composition*										
Body mass (kg)	76.8 (15.4)	77.0 (16.4)	82.1 (13.9)	81.7 (15.1)	81.7 (16.3)	81.7 (16.0)		.224 (.399)	−.624 (.703)	−.243 (.564)
Waist circumference (cm)	91 (12)	91 (12)	96 (13)	97 (13)	99 (12)	98 (13)		−.550 (.897)	1.550 (1.553)	.121 (1.253)
Fat mass (kg)	25.2 (6.4)	25.8 (6.6)	27.8 (8.5)	27.9 (8.3)	29.6 (8.1)	29.8 (8.7)		.625 (.721)	−.487 (1.269)	−.480 (1.019)
Lean body mass (kg)	50.8 (13.3)	52.0 (14.2)	54.4 (10.7)	53.8 (11.2)	52.1 (11.8)	51.9 (11.7)		1.218 (.612)	−1.756 (1.077)	−1.382 (0.865)
*Blood markers*										
CRP (mg/l)	3.07 (5.72)	2.53 (4.51)	4.29 (3.72)	2.44 (1.69)	8.15 (16.7)	6.22 (15.6)		−.535 (1.454)	−1.309 (2.609)	−1.404 (2.112)
IL-6 (pg/ml)	2.69 (1.71)	3.07 (4.19)	2.75 (1.37)	2.88 (2.31)	6.60 (8.10)	4.83 (7.07)		−.375 (.896)	.249 (1.552)	2.140 (1.302)
Glucose (mmol/l)	5.49 (.94)	6.08 (1.33)	5.18 (.54)	5.40 (.47)	5.44 (.80)	5.13 (.63)		.590 (.214)	−.369 (.371)	−.892 (.307)*
HDL (mmol/l)	1.58 (.43)	1.56 (.41)	1.31 (.25)	1.30 (.30)	1.46 (.37)	1.51 (.37)		−.018 (0.052)	.004 (.091)	.070 (.075)
LDL (mmol/l)	3.16 (.78)	3.13 (.94)	3.02 (.95)	3.01 (.87)	2.62 (.97)	2.55 (.96)		−.029 (.104)	.027 (.180)	−.041 (.1459)
Triglycerides (mmol/l)	1.41 (.60)	1.63 (1.05)	1.63 (.53)	1.84 (.65)	1.21 (.54)	1.47 (.78)		.223 (.150)	−.009 (.260)	.037 (.215)
Total cholesterol (mmol/l)	6.14 (1.09)	6.32 (1.29)	5.96 (1.10)	6.16 (.99)	5.29 (1.09)	5.53 (1.21)		.173 (.159)	.025 (.276)	.069 (.228)

WHM: Wim Hof Method; UC: usual care; *N*: number of participants; β: beta coefficient; SE: standard error; Pre: pretreatment assessment; Post: posttreatment assessment; FVC: forced vital capacity; FEV-1: forced expiratory volume in 1 s; FIV-1: forced inspiratory volume in 1 s; PEF: peak expiratory flow; PIF: peak inspiratory flow; BP: blood pressure; CRP: C-reactive protein; IL-6: Interleukin 6; HDL: high-density lipoprotein; LDL: low-density lipoprotein.

* The values in bold and marked with asterisks indicate the significantly different changes over time between the two groups.

### Primary Outcome

Twenty-two participants (42%) had mental health issues (*n* = 12) or severe mental health issues (*n* = 10) at baseline. Twelve percent of the total group shifted to a worse mental health category, 71% remained in the same category, and 17% shifted to a better category following the intervention phase. The three groups did not differ significantly in mental health status at pretreatment (*P* = 0.97) nor in the change in category over time (*P* = 0.90).

**Figure 2.**
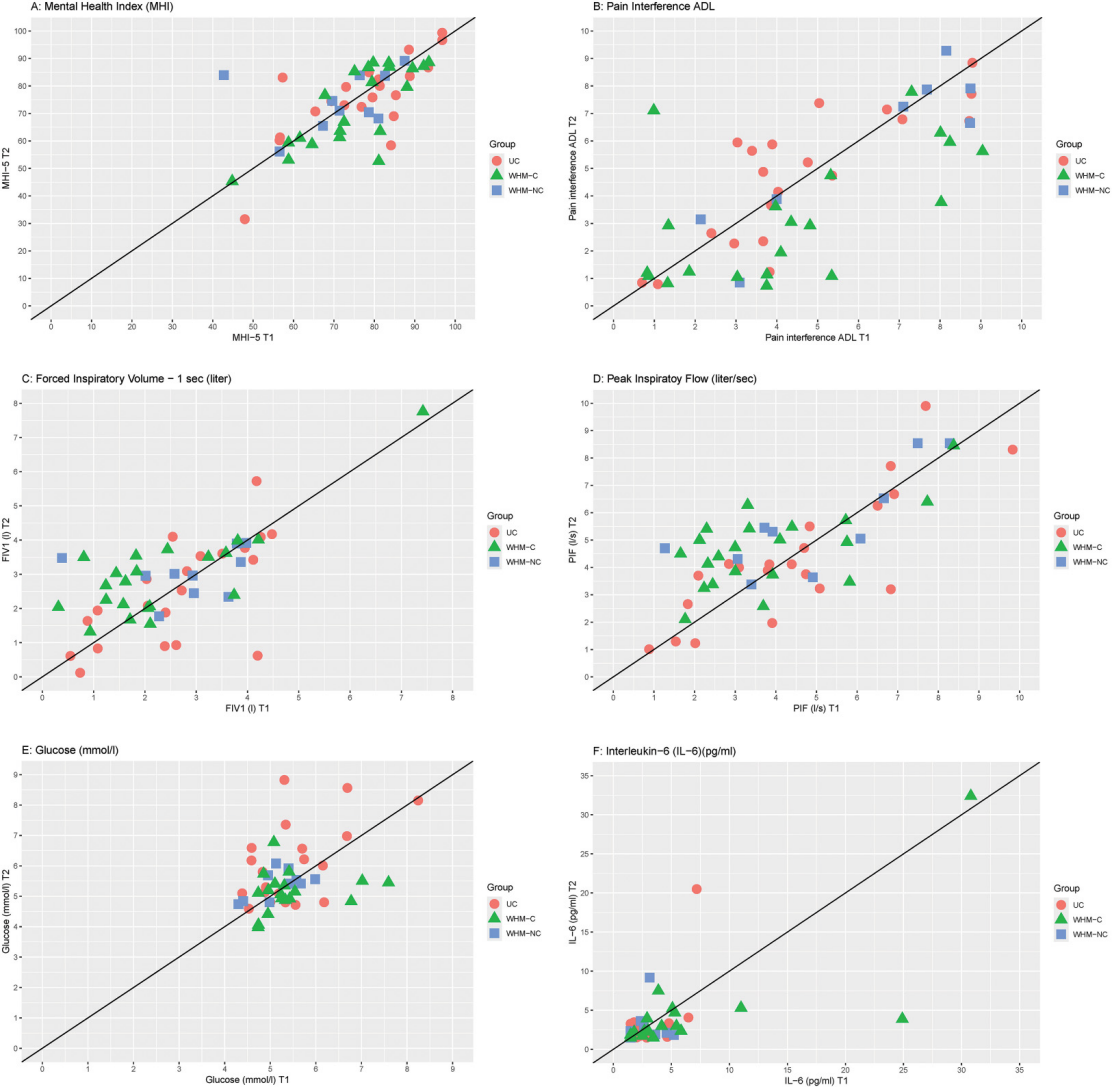
Overview of the individual data at the pretest (T1, *x*-axis) and posttest (T2, *y*-axis) for (A) mental health index (MHI-5), (B) pain interference in activities of daily life (ADL), (C) forced inspiratory volume in 1 s (FIV1), (D) peak inspiratory flow (PIF), (E) glucose concentration, and (F) interleukin-6 (IL-6) concentration. UC: Usual Care group; WHM-C: Wim Hof Method intervention group *with* cold exposure; WHM-NC: Wim Hof Method intervention group without cold exposure. The diagonal line is the line of identity (loi): when the dot is on this line it indicates that the pre and post values are exactly the same. Favorable changes are seen when the dots are on the left side of the loi for the MHI-5, FIV1, and PIF, and on the right side of the loi for pain interference in ADL, glucose, and IL-6 concentration.

The continuous outcome, Mental Health Index-5, did not show a significant change over time between the three groups (Table 2, Figure 2A).

### Psychological Outcomes

No significant differences in change over time were observed among the three groups, except for the impact that pain had on performing daily activities in the past week (Table 2; Figure 2B). The group practicing the Wim Hof Method with cold exposure showed a reduced impact of pain on daily activities, compared to the usual care group.

### Physical Health Outcomes

Table 3 presents the physical health outcomes for the three groups at both pre- and posttreatment assessments. Inspiratory parameters—forced inspiratory volume in 1 s and peak inspiratory flow—significantly increased over time (Figure 2C and 2D), with the Wim Hof Method including cold exposure demonstrating an improvement in contrast to usual care. Furthermore, the Wim Hof Method with cold exposure showed a significant reduction in glucose concentration relative to the usual care group (Figure 2E). No significant effects were found for expiratory parameters, blood pressure, body composition, inflammatory markers such as interleukin-6 (Figure 2F), or lipid concentration. Sensitivity analyses for C-reactive protein and interleukin-6 yielded comparable results, that is, no significant effects.

### Adherence

Table 4 shows adherence to the number of breath cycles, the number of breaths per cycle, and cold showering during the home sessions between the first and last group session. In weeks 1 and 2, few participants took a cold shower since they were instructed to start with cold cloths rather than showering.

**Table 4.** Overview of the adherence of the breathing exercise (number of breath cycles, breaths per cycle) and cold exposure protocol followed by the WHM-intervention groups between the first and last group session (*N* = 31 for breathing exercises and *N* = 21 for cold exposure).

	According to protocol	Week 1	Week 2	Week 3	Week 4	Week 5	Week 6
Day 1	Number of breath cycles, *n* (%)	22 (71%)	27 (87%)	27 (87%)	26 (83%)	21 (68%)	21 (68%)
	Number of breaths, *n* (%)	25 (81%)	28 (90%)	28 (90%)	24 (77%)	21 (68%)	23 (74%)
	Took a cold shower, *n* (%)	1 (5%)	4 (19%)	16 (75%)	15 (71%)	15 (71%)	13 (62%)
Day 2	Number of breath cycles, *n* (%)	25 (81%)	24 (77%)	29 (94%)	24 (77%)	20 (65%)	20 (65%)
	Number of breaths, *n* (%)	26 (84%)	26 (84%)	30 (97%)	25 (81%)	23 (74%)	18 (58%)
	Took a cold shower, *n* (%)	1 (5%)	4 (19%)	17 (81%)	15 (71%)	16 (76%)	12 (57%)
Day 3	Number of breath cycles, *n* (%)	24 (77%)	25 (81%)	25 (81%)	20 (65%)	25 (81%)	20 (65%)
	Number of breaths, *n* (%)	25 (81%)	25 (81%)	26 (84%)	23 (74%)	25 (81%)	23 (74%)
	Took a cold shower, *n* (%)	2 (10%)	4 (19%)	14 (67%)	18 (86%)	16 (76%)	16 (76%)
Day 4	Number of breath cycles, *n* (%)	27 (87%)	25 (81%)	26 (84%)	22 (71%)	20 (65%)	21 (68%)
	Number of breaths, *n* (%)	29 (94%)	26 (84%)	27 (87%)	22 (71%)	20 (65%)	20 (65%)
	Took a cold shower, *n* (%)	3 (14%)	4 (19%)	12 (57%)	15 (71%)	14 (67%)	12 (57%)
Day 5	Number of breath cycles, *n* (%)	26 (84%)	25 (81%)	28 (90%)	22 (71%)	22 (71%)	19 (61%)
	Number of breaths, *n* (%)	27 (87%)	24 (77%)	29 (94%)	22 (71%)	20 (65%)	18 (58%)
	Took a cold shower, *n* (%)	2 (10%)	5 (24%)	16 (76%)	19 (90%)	15 (71%)	11 (52%)
Day 6	Number of breath cycles, *n* (%)	25 (81%)	25 (81%)	26 (84%)	23 (74%)	19 (61%)	14 (45%)
	Number of breaths, *n* (%)	28 (90%)	25 (81%)	28 (90%)	21 (68%)	21 (68%)	14 (45%)
	Took a cold shower, *n* (%)	3 (14%)	5 (24%)	16 (76%)	14 (67%)	14 (67%)	13 (62%)
Day 7	Number of breath cycles, *n* (%)	24 (77%)	23 (74%)	26 (84%)	23 (74%)	22 (71%)	22 (71%)
	Number of breaths, *n* (%)	24 (77%)	25 (81%)	27 (87%)	24 (77%)	21 (68%)	18 (58%)
	Took a cold shower, *n* (%)	2 (10%)	5 (24%)	15 (71%)	13 (62%)	15 (71%)	11 (52%)
	**Weekly mean adherence to protocol**						
	Number of breath cycles (%)	80%	80%	86%	74%	69%	63%
	Number of breaths (%)	85%	83%	90%	74%	70%	62%
	Took a cold shower (%)	10%	21%	72%	74%	73%	60%

Participants were instructed to perform 3 cycles of 30 breaths during weeks 1–3, and 4 cycles of 40 breaths during weeks 4–7. Cold exposure progressed in stages: during weeks 1–2, participants used cold cloths only (no shower); in weeks 3–4, they began cold showers starting at 30 s, increasing by 10 s with each session; and in the final weeks (5–7), they were instructed to take cold showers lasting 150 s.

### Adverse Events / Participant Experiences

No serious adverse events occurred during the study. A range of experiences were reported during the breathing exercises and cold exposure, though not all were perceived as negative.

Participants reported tingling sensations in the head, upper body, arms, and legs (*N* = 8); headaches (*N* = 5), lightheadedness (*N* = 3), burning below the lesion level (*N* = 3), nerve pain (*N* = 3), and spasms (*N* = 3). Additional symptoms, each mentioned once, included anxiety, cold extremities, chest pressure, unpleasant sensations, foot cramps, throat and head pressure, and mild shocks in the lower limbs.

Reported experiences related to cold exposure in the Wim Hof Method group were as follows: feeling cold all day after shower (*N* = 3); painful cold showers (*N* = 2); spasms during or after cold showers (*N* = 2); cold/stiff muscles and cramps (*N* = 1); and difficulty breathing (*N* = 1). Increased pain, reduced sleep quality, and severe pain were each mentioned by one participant in the Wim Hof Method with cold exposure group, without being specifically attributed to the breathing exercises or cold exposure. Importantly, no cases of autonomic dysreflexia were reported during the cold showers or the optional ice bath (*N* = 14 out of 21).

### Exit Interview

The Wim Hof Method intervention received an excellent rating on a 0–10 scale from both the group without cold exposure (8.8 ± 0.8, *N* = 10) and the group with cold exposure (8.7 ± 1.0, *N* = 21). Three (30%) participants in the group without cold exposure and 10 (48%) in the group with cold exposure responded (very) certain to the statement “The training has led to a better understanding of my reactions to my spinal cord injury.” To the statement “The exercises have helped me cope with the effects of the spinal cord injury” responded 5 (50%) participants of the group without cold exposure and 8 (38%) of the group with cold exposure (very) certain. Table 5 shows the answers to the open questions in the exit interview and indicated the various positive effects that were experienced.

**Table 5.** Results in-person exit interview.

**Can you specify which effects of the spinal cord injury you handle differently?**	**N**	**Name 1-2 aspects that are valuable to you and that you will continue to use**	**N**
Breathing exercise lead to:		Breathing exercises in general	19
More relaxed	5	Cold showering in general	6
Better dealing with pain	2		
Falling asleep faster	1	Breathing exercises:	
Warming up faster when cold	1	to relax	3
		when waking up	2
Breathing & cold exposure lead to:		for focussing	2
More influence on health	2	to fall asleep	1
Feeling fitter	2	in addition to mindfulness	1
		to use during endurance sport	1
More consciously engaged with pain,	2	Breathing exercises & cold showering	1
sleep, and mood		Retention phase: feels like body is	1
More energy	2	healing	1
Better, and more aware of my, breathing	1	Taking an ice bath once a week	1
More in contact with legs and feet	1	Session with instructor, had greater	2
Less medication	1	impact (on my emotions)	
Mindset to step outside my comfort zone	1	App group to keep motivating each	1
I feel better overall	1	other	

## Discussion

This randomized controlled trial assessed the efficacy and feasibility of an accessible, predominantly home-based 7-week intervention combining breathing exercises, mindset training, and incremental cold exposure for individuals with chronic spinal cord injury. Regarding efficacy, changes in the primary outcome measure—mental health—did not differ significantly between the three groups. Nevertheless, inspiratory parameters and fasting glucose concentration improved more in the Wim Hof Method with cold exposure than in the usual care group. This intervention group also reported a greater reduction in pain-related interference with daily activities.

Based on adherence rates and exit interview responses, the intervention was generally well-tolerated and positively received across a wide range of lesion levels. This suggests its potential suitability for diverse subgroups within the spinal cord injury population. However, the Wim Hof Method without cold exposure experienced a substantial number of dropouts. These could not be directly attributed to the intervention, as the group with cold exposure also followed that part of the protocol.

Similar to our pilot trial—which used the same instructors and procedures but had a shorter duration (4 weeks)^7^—neither the Wim Hof Method with nor without cold exposure improved mental health. This lack of improvement contrasts with mental health results from other trials investigating the Wim Hof Method in healthy volunteers^8,25^ or patients with axial spondyloarthritis.^5^ In some cases,^7,8^ the presence of a control group in the current study may explain the difference in outcomes. Effects observed in a pre–post study often disappear in a randomized controlled trial because the control group shows that the change is not due to the intervention itself, but to time, expectation, regression to the mean, or other non-specific factors. However, randomized controlled trials investigating other populations^5,25^ have shown improvements in perceived stress and mental health, respectively.

Notably, Buijze et al.^5^ reported that a higher volume of cold exposure, including weekly cold baths and daily showers from the start of the study, may be necessary to achieve mental health benefits. In comparison, the present study included a 2-week run-in period, cold showers only on scheduled days (not always daily), and a single optional ice bath. This lower dose of cold exposure may have been insufficient to elicit psychological effects. More speculatively, some of the mental health benefits of the Wim Hof Method are thought to be mediated by sympathetic activation during cold exposure.^26^ Since spinal cord injury is associated with impaired sympathetic responses to physiological stress,^27^ the effects of the Wim Hof Method on mental health outcomes may differ in this population.

Interestingly, the Wim Hof Method with cold exposure led to a reduced perceived impact of pain during daily activities, even though pain intensity itself did not change. Building on meditation literature,^28^ it may be that the mindfulness involved in the breathing exercises and cold exposure helped participants better cope with chronic pain in everyday life. However, the self-reported mindfulness did not change in either of our intervention groups. Alternatively, the acute stress response to cold exposure, likely including elevations in β-endorphin concentration, has analgesic effects as shown by an increased pain threshold immediately following a cold pressor test.^29,30^ This repeated response following each cold exposure may explain improvements in pain interference without parallel changes in mindfulness.

On the other hand, the lack of change in pain intensity aligns with a systematic review^31^ showing that only one out of the four mindfulness interventions in individuals with spinal cord injury led to a reduction in pain. Notably, this effect was only observed in the group practicing the Wim Hof Method with cold exposure, suggesting a potential synergistic benefit of combining its components. Although the small sample size in the group without cold exposure may have limited statistical power, this finding supports earlier suggestions that the combination of breathing exercises and cold exposure is more effective than either component alone.^26^

Pulmonary function is often impaired following spinal cord injury, depending on the level and completeness of the lesion. This can hinder daily activities^32^ and, particularly in persons with tetraplegia, reduced coughing ability may lead to mucus build-up and increased risk of pneumonia. While expiratory function is directly linked to coughing, inspiratory function also plays a role.^33^ Therefore, the observed improvements in forced inspiratory volume in one second and peak inspiratory flow are clinically meaningful. For instance, Postma et al. found a significant relationship between pulmonary function and quality of life in persons with spinal cord injury 5 years after rehabilitation discharge.

Interestingly, unlike our pilot trial^7^ where breathing exercises alone improved pulmonary function, only the group practicing the Wim Hof Method with cold exposure showed significant improvements in this randomized controlled trial. We do not have a clear explanation for the lack of improvement in the Wim Hof Method without cold exposure group, as this group is similar in terms of lesion-level distribution (tetraplegia vs. paraplegia), lesion completeness (AIS A–D), and age compared with the pilot study group. The discrepancy may be explained by the fact that the improvement observed in the pilot study was relatively small, and that this effect is no longer significant now that the comparison is made against usual care rather than a pre- vs. postintervention comparison.

Another explanation may be a potential synergistic effect of combining cold exposure and breathing exercise. Cold exposure may increase respiratory muscle demand, especially when performed immediately after breathing exercises. A similar synergistic effect has previously been reported for the psychological outcome perceived stress.^26^ The breathing exercises and cold exposure in that study, when applied individually, did not produce substantial effects on perceived stress. In contrast, the group that received the combination of both components showed a medium- to large-sized improvement in perceived stress compared to the control group. There is little comparative evidence on pulmonary function following the Wim Hof Method. In able-bodied individuals, breathing economy during exercise did not improve following the method with cold exposure.^34^

Although regular exercise can help preserve pulmonary function,^35^ only 12–29% of individuals with spinal cord injury meet recommended guidelines—often due to limited access to equipment or physical capacity.^35,36^ In this context, the Wim Hof Method may offer a more feasible alternative to improve pulmonary function, especially given its accessibility and home-based nature.

Nonexercise interventions to improve pulmonary function include inspiratory muscle training and yoga-based breathing exercises. The inspiratory parameters—forced inspiratory volume in 1 s and peak inspiratory flow—showed improvements of 23–27% in the Wim Hof Method with cold exposure group (based on the absolute values in Table 3). These improvements are comparable to, or even greater than, those reported in other populations following respiratory muscle training (10–16%).^37,38^ Given the robustness of these findings—both in this trial and our earlier pilot^7^—the Wim Hof Method with cold exposure should be considered an accessible intervention to improve pulmonary function in preventative care for individuals with spinal cord injury. Importantly, the breathing exercises were also feasible for individuals with motor complete tetraplegia, as no hand function is required—unlike traditional inspiratory muscle training devices. This makes the Wim Hof Method highly accessible.

Emerging evidence supports the role of repeated cold exposure in improving aspects of metabolic health.^39,40^ In this study, fasting glucose concentrations reduced significantly in the Wim Hof Method with cold exposure group versus usual care. This finding aligns with previous studies showing improved insulin sensitivity after 10 days of daily cold exposure in an environmental chamber in patients with type 2 diabetes mellitus^10^ and improved lipid profile after 5 months of cold water immersion in able-bodied individuals.^41^ Interestingly, the cold exposure in those studies was more intense and of longer duration than in the current trial. It is therefore unlikely that the cold exposure currently employed was sufficient to activate brown adipose tissue,^10,39^ which is often cited as a mechanism for metabolic benefits. It should also be acknowledged that other metabolic markers—including inflammatory markers, lipids, blood pressure, and or body composition—did not improve following either intervention.

Overall, the Wim Hof Method intervention was rated highly by participants. Nearly half reported an improved understanding of their spinal cord injury and better coping with its effects—an encouraging outcome given the average time since injury of 20 years.

Some participant experiences were expected, such as tingling in head, upper body, arms, and legs, as well as headache and lightheadedness. These effects are consistent with the deep consecutive inhalations and relaxed exhalations used during the intervention.^42^ While many participants described the sensations positively—such as a tingling sensation in their paralyzed legs—others found them uncomfortable, as they triggered spasms and pain. Consequently, three participants discontinued part of the intervention. Importantly, negative experiences could not be linked to specific participant characteristic (e.g., motor complete tetraplegia), suggesting that individual tolerance and perception play a key role in determining the intervention's suitability.

Key strengths of this study include the long duration compared with several other studies in the field, the systematic adherence monitoring and the inclusion of three study arms, allowing for evaluation of the additive value of cold exposure.

Several limitations should be noted as well. The study groups were relatively small, and a large proportion of participants in the intervention group without cold exposure withdrew, reducing statistical power. Unfortunately, the participants who dropped out also declined the posttreatment assessment, so we could not include their posttreatment data in a standard linear regression of change for an intention-to-treat analysis. Given the extent and pattern of missing data, estimates of the posttreatment effect for dropouts would be too uncertain to base firm conclusions on. Additionally, some participants dropped out or did not adhere to the protocol due to treatment-related reasons. All these factors may have influenced the outcomes and should be considered when interpreting the results. Moreover, although the overall proportions were comparable between groups, the Wim Hof Method with cold exposure group included a higher number of participants with a motor-complete lesion (ASIA A-B). This may have influenced the breathing exercises, which could have been less deep, and subsequently may have affected various outcomes. Lastly, the large number of statistical comparisons made within our small group may have led to statistically significant differences by chance alone. We therefore acknowledge the potential for a Type I error due to multiple analyses without correction of the alpha value.

Although somewhat inherent to home-based trials, the cold showers were not as standardized and cold (14.6 °C) as for example a laboratory-based ice bath would have been. However, this enhances the representativeness of the results when implementing the intervention in a home setting. In addition, adherence declined after week 3, highlighting the need for strategies to maintain engagement. Lastly, lack of a long-term follow-up is also a weakness of this study.

Online home-based group sessions may be considered when implementing this intervention in clinical practice or in future studies. These may be easier to adhere to than individual home-based sessions, and evidence suggests that online sessions can be effective.^8^

Overall, the accessibility of the intervention makes it a promising option for preventative care, especially for improving pulmonary function. Improvements in inspiratory parameters suggest the method may help mitigate respiratory complications. Its feasibility without requiring hand function makes it suitable for a broader range of individuals. While many participants reported positive sensations, others experienced discomfort, spasms, or pain. Clinicians should assess individual tolerance and monitor responses closely.

Future studies should aim for larger, more balanced groups to improve statistical power and reduce bias. It would be interesting to investigate whether more frequent and intense cold exposure yields stronger effects on mental health and metabolic outcomes, as suggested by previous studies. Given the role of sympathetic activation in mediating psychological and metabolic effects,^9^ future trials should include physiological markers—such as heart rate variability—to better understand mechanisms—especially in individuals with spinal cord injury who may have impaired autonomic function. A recommendation is to stratify participants also by baseline tolerance to cold and breathing exercises to identify subgroups most likely to benefit. Finally, it remains important to assess whether the benefits are sustained over time and if continued practice leads to further improvements.
